# Determinants of Parental Vaccine Hesitancy During the COVID-19 Era in Saudi Arabia: A Cross-Sectional Survey

**DOI:** 10.7759/cureus.66129

**Published:** 2024-08-04

**Authors:** Khaldoon Aljerian, Hamad AlMadi, Nawaf H AlMadi, Abdulaziz AlKahtani, Hussam AlGhamdi, Ahmed Al-Ghamdi, Abdulaziz Al-Assaf, Abdulaziz AlSubaie, Mohamad-Hani Temsah

**Affiliations:** 1 Pathology, King Saud University College of Medicine, Riyadh, SAU; 2 Emergency Medicine, Prince Sultan Military Medical City, Riyadh, SAU; 3 Medical Student, King Saud University College of Medicine, Riyadh, SAU; 4 Pediatrics, Pediatric Intensive Care Unit, King Saud University Medical City, King Saud University, Riyadh, SAU

**Keywords:** vaccine, parents’ hesitancy, saudi arabia, routine childhood vaccination, covid-19 era

## Abstract

Background

Despite the success of childhood vaccination in reducing vaccine-preventable diseases (VPDs), vaccine hesitancy remains a significant challenge in several countries, such as Saudi Arabia, both during and beyond the COVID-19 era. Furthermore, the pandemic may have impacted vaccine hesitancy trends, potentially affecting parents’ intentions to adhere to scheduled childhood vaccination programs.

Aim

This article aims to assess the extent of parents’ hesitancy toward childhood vaccination, determine if it increased or decreased due to the COVID-19 pandemic, highlight the factors and determinants that influenced this hesitancy, whether positively or negatively, during the COVID-19 era, and estimate the acceptance of COVID-19 vaccination in relation to the acceptance of scheduled childhood vaccination.

Methods

A cross-sectional study was conducted in Saudi Arabia through a snowball sampling technique. Data were collected between September 2022 and October 2022 using an online survey using Google Forms. The inclusion criteria were parents or guardians in Saudi Arabia with a child up to 18 years of age. Responses were analyzed using SPSS V25 (IBM Corp., Armonk, NY, US), with chi-square tests and logistic regression performed to compare hesitancy and vaccination status.

Results

Among the 1,209 parents and care providers who participated, the prevalence of parents’ vaccine hesitancy was 374 (30.9%). The educational level of the parents was not significantly associated with hesitancy status (p 0.490). The most refused vaccine was Mpox (345; 28.5%), whereas the one that caused the most hesitancy was the COVID-19 vaccine (352; 29.1%). Regarding the parents’ concerns, the main reason for their hesitancy was the influence of their negative perceptions from social media content, including false or misleading information and negative allegations about vaccines, reported by 449 (18.98%) of the participants. Logistic regression analysis indicated that negative social media perceptions significantly increased the likelihood of vaccine hesitancy (OR = 2.15, 95% CI = 1.78-2.60, p < 0.001).

Conclusion

Our study highlights the prevalence of parental vaccine hesitancy during the COVID-19 era; the most significant hesitancy was observed toward the COVID-19 vaccine, and the mpox vaccine was the most rejected. Negative social media was the main reason for parental hesitancy; public health efforts should focus on providing accurate and easily accessible information through educational campaigns on social media and other platforms.

## Introduction

Vaccination is one of the most effective and safe public health interventions and is considered to be the greatest achievement in the last century, as it is estimated to save 2 to 3 million lives each year. Despite its success, a vaccine confidence gap is emerging in all developed and developing countries [1-4].

In 1979, the Kingdom of Saudi Arabia (KSA) adopted the Expanded Program on Immunisation (EPI), which had been previously launched by the WHO [5]. In 2019, the program succeeded in improving the immunization coverage of measles by 96%. Moreover, a noticeable reduction of more than 90% was observed in the incidence of vaccine-preventable diseases (VPDs) [6].

The term ‘vaccine hesitancy’ was defined by the WHO in 2012 as a refusal or delay of vaccination when such services are available; the organization listed it as one of the top 10 health threats of 2019 [7]. Vaccine hesitancy is complex and is influenced by various factors, such as location, time, and vaccine types, and can be categorized into three main subtypes: confidence, complacency, and convenience [7,8]. Confidence relates to the trust in the clinical effectiveness and safety profile of vaccines, the healthcare system that delivers them, and the motivations of policymakers. Complacency occurs when the perceived risks of acquiring vaccine-preventable diseases are low, and therefore, vaccination is not seen as a necessary preventive measure. Convenience involves the public accessibility of vaccines, including the availability, cost affordability, and appeal of immunization services​ [7,8]. Parental hesitancy toward scheduled childhood vaccination is an important issue that needs to be addressed because effective control of VPDs in children requires high rates of timely vaccination [9].

Before the COVID-19 era, parental vaccine hesitancy was centered around different vaccinations, such as influenza; measles, mumps, rubella (MMR), hepatitis B virus (HBV), and others [10,11]. Negative information on social media might have increased concerns about vaccine safety [12]. The recent pandemic may have influenced current vaccine hesitancy trends, potentially exacerbating existing concerns and introducing new factors specific to COVID-19 vaccines. However, it is important to recognize that this is not a unidirectional cause-effect relationship, as multiple factors can interact to influence vaccines [7].

The COVID-19 pandemic has awakened concerns about vaccine hesitancy, and understanding whether this hesitancy extends beyond the COVID-19 vaccine to other scheduled childhood vaccines is crucial. To address this issue, this study aimed to assess the impact of the COVID-19 pandemic on parental hesitancy toward childhood vaccination in KSA and the factors that might have contributed to it. Additionally, we explored the acceptance of the COVID-19 vaccine among parents and its relationship to their acceptance of scheduled childhood vaccinations.

## Materials and methods

This observational cross-sectional study was conducted in Saudi Arabia. The inclusion criteria were being a parent or guardian residing in KSA during the study period, having a child (up to 18 years of age), and, after being informed about the study aims, agreeing to voluntarily participate in the research. The exclusion criteria were having no children or living outside KSA at the time of data collection.

The minimal sample size was estimated to be 385, considering a confidence interval (CI) of 95%, a margin error of 5%, a population proportion of 50%, and an estimated population of 3,141,651, which is the number of Saudis married - a figure taken from the General Authority for Statistics demography survey [13]. Participants received an online questionnaire consisting of 30 categorical items. These items included demographic questions, Likert scale assessments, and yes/no questions to capture a range of responses regarding vaccine hesitancy and related factors. The questionnaire was formulated based on reviewing previous WHO questionnaires from similar studies with some adjustments to better fit our objectives [14]. The data were collected between September and October 2022 through a convenience sampling technique. Approval for the study was obtained from the Institutional Research Board (IRB # E-22-7049) of King Saud University, Riyadh, KSA.

Data collection

A snowball sampling method was used through two main social media platforms in KSA (Twitter and WhatsApp) as a way of rapid subject recruitment. Respondents were asked to forward the questionnaire to others in the target population. Only complete responses were included in the analysis to ensure data integrity. The survey link was distributed via posts and messages on Twitter and WhatsApp, ensuring accessibility and ease of participation. Non-response rates were reminded once to encourage completion. Incomplete responses were excluded, and the survey link could be completed only once from the same device to prevent duplicate responses.

Statistical analysis

Statistical analysis was performed using the Statistical Package for the Social Sciences version 25 (SPSS V25; IBM Corp., Armonk, NY, US). Frequencies and percentages were used to summarize the categorical variables. Comparing the yes/no answers in terms of hesitancy status between yes and no was performed using the chi-square test, and comparing the answers related to vaccination status (i.e., fully immunized, partially immunized, or unimmunized) was performed using the chi-square test too. Logistic regression with a 95% confidence interval was performed.

## Results

Table 1 lists the sociodemographic details of the respondents (N=1209), with almost half of the surveyed parents 626 (51.8%) mothers, and most of them (894; 73.9%) had completed a bachelor’s or postgraduate degree. The educational level did not show a statistically significant association with vaccination and hesitancy status. Parents with bachelor's degrees or postgraduate degrees were not found to be more vaccine-hesitant than parents with school degrees (p-value = 0.490). Regarding childhood vaccine hesitancy, 374 (30.9%) of the parents expressed doubts or hesitancy about vaccinating their children. Among the participating parents, the survey revealed that only 282 (23.2%) of children remained unvaccinated while 211 (17.5%) of children were not fully immunized for their age.

**Table 1:** Distribution of demographics characteristics of study subjects (N=1209)

Variables	N (%)
Participants	
Father	308 (25.5)
Mother	626 (51.8)
Another member of the family	275 (22.7)
Education level of participants	
School	248 (20.5)
Associate degree/ diploma	24 (2.0)
Bachelor’s or postgraduate	894 (73.9)
Masters/PhD	43 (3.5)
Participant age (year)	
18-24	173 (14.3)
25-34	250 (20.7)
35-44	416 (34.4)
45-54	271 (22.4)
55-64	82 (6.8)
> 65	17 (1.4)
Number of children under care of the participants	
Mean (SD)	3.16 (2.19)
Min- max	0-10
Age of the youngest child under care of the participants (year)	
Mean (SD)	5.55(5.19)
Min- max	0-30
Immunization status of the youngest child in the care of the participants	
Fully immunized	716 (59.2)
Partly immunized	211 (17.5)
Unimmunized	282 (23.2)
Hesitancy of parents to vaccinate their children	
Yes	374 (30.9)

Table 2 presents the levels of parental hesitancy toward different vaccines. The COVID-19 vaccine had the highest hesitancy rate, with 352 (29.1%) of parents expressing reluctance toward it. Following closely was the updated COVID-19 Omicron BA.4 and BA.5 vaccine, with 335 (27.7%) expressing hesitancy and 318 (26.3%) refusing. The Mpox vaccine had the highest refusal rate, with 345 (28.5%) of parents declining it. For all other vaccines, the majority of parents did not express hesitancy or refusal.

**Table 2:** Distribution of study subject's responses toward hesitancy, refusal, and no refusal or hesitation according to type of vaccine

Vaccine		Hesitant	Refused	Did Not Refuse or Hesitate
COVID-19		352 (29.1)	298 (24.6)	559 (46.2)
Updated COVID-19 Omicron BA.4 and BA.5		335 (27.7)	318 (26.3)	556 (46.0)
Mpox		183 (15.1)	345 (28.5)	681 (56.3)
Chickenpox	108 (8.9)		143 (11.8)	958 (79.2)
Hemophilus influenza b		142 (11.7)	133 (11.0)	934 (77.3)
Hepatitis B		83 (6.9)	84 (6.9)	1042 (86.2)
Human papilloma virus		149 (12.3)	135 (11.2)	925(76.5)
Influenza		163 (13.5)	165 (13.6)	881 (72.9)
Polio		62 (5.1)	64 (5.3)	1083 (89.6)
Measles		63 (5.2)	61 (5.0)	1085 (89.7)
Meningococcal		80(6.6)	73 (6.0)	1056 (87.3)
Mumps		86 (7.1)	85 (7.0)	1038 (85.9)
Rubella		79 (6.5)	78(6.5)	1052 (87.0)
29 “Pentavalent” or other combination infant		113 (9.3)	94 (7.8)	1002 (82.9)
Pneumococcal		120 (9.9)	106 (8.8)	983 (81.3)
Rotavirus		134 (11.1)	113 (9.3)	962 (79.6)
Tetanus, diphtheria, pertussis		96 (7.9)	82 (6.8)	1031 (85.3)

Table 3 illustrates the participants' attitudes towards vaccines. A significant portion of parents expressed that ‘new vaccines carry more risks than older vaccines’, with 368 (30.4%) strongly agreeing and 388 (32.1%) agreeing. Furthermore, 442 (36.6%) of parents were concerned and 367 (30.4%) were strongly concerned about the potential for serious adverse effects from vaccines. In addition, 313 (25.9%) of parents strongly agreed and 380 (31.4%) agreed that their children do not need vaccines for diseases that are no longer common. 

**Table 3:** Distribution and comparison of study subject's responses toward vaccines

Vaccine attitude scale	Strongly disagree	Disagree	Agree	Strongly agree
Childhood vaccines important for my child's health	38 (3.1)	36 (3.0)	413 (34.2)	718 (59.4)
Childhood vaccines are effective	38 (3.1)	45 (3.7)	458 (37.9)	668 (55.3)
Having my child vaccinated is important for the health of others in my community	42 (3.5)	50 (4.1)	426 (35.2)	691 (57.2)
All childhood vaccines offered by the government program in my community are beneficial	44 (3.6)	76 (6.3)	393 (32.5)	696 (57.6)
New vaccines carry more risks than older vaccines	368 (30.4)	300 (24.8)	388 (32.1)	368 (30.4)
The information I receive about vaccines from the vaccine program is reliable and trustworthy.	63 (5.2)	148 (12.2)	489 (40.4)	509 (42.1)
Getting vaccines is a good way to protect my child/children from disease.	49 (4.1)	49 (4.1)	479 (39.6)	632 (52.3)
Generally, I do what my doctor or health care provider recommends about vaccines for my child/children.	42 (3.5)	61 (5.0)	494 (40.9)	612 (50.6)
I am concerned about the serious adverse effects of vaccines.	173 (14.3)	227 (18.8)	442 (36.6)	367 (30.4)
My children do not need vaccines for diseases that are not common anymore.	268 (22.2)	248 (20.5)	380 (31.4)	313 (25.9)

Table 4 shows concerns reported by those hesitant to get their children vaccinated. A total of 449 (18.98%) of the parents heard or read negative media content, which is the most common reason parents are hesitant. This is followed by 379 (16.02%) hesitant parents who did not think the vaccine was safe/were concerned about side effects, which is the second most common reason parents are hesitant. Did not think it was needed 273 (11.54%) and someone else told me that the vaccine was not safe 155 (6.55%) are other common reasons parents are hesitant.

**Table 4:** Distribution of study subject's responses toward their concerns regarding getting their children vaccinated

Concerns	N (%)
Didn’t hesitate	471(19.92)
Did not think it was needed	273 (11.54)
Heard or read negative media	449 (18.98)
Did not know where to get vaccination	46 (1.94)
Had a bad experience or reaction with a previous vaccination	96 (4.06)
Did not know where to get good/reliable information	127 (5.37)
Had a bad experience with a previous vaccinator/health clinic	28 (1.18)
Someone else told me they/their child had a bad reaction	136 (5.75)
Did not think the vaccine was effective	126 (5.33)
Someone else told me that the vaccine was not safe	155 (6.55)
Did not think the vaccine was safe/ concerned about side effects	379 (16.02)
Fear of needles	36 (1.52)
Religious reasons	7 (0.29)
Other beliefs/folk medicine	36 (1.55)

The multivariable logistic binary regression analysis was applied to people's perceived hesitance toward the children's vaccination dichotomized score to gain more insight into what may explain people's hesitance toward children's vaccines. The resulting analysis findings (Table 5) showed that the respondent (being a parent or other relative) did not correlate significantly with people's odds of being reluctant to children's vaccinations (p-value=0.415). But people aged >=45 years were found to be significantly less reluctant (i.e., less hesitant) to children's vaccinations (23.5% times less ) compared to people aged <45 years on average (p-value<0.001). Also, the yielded analysis findings showed that the evermarried people were found to be significantly more predicted to be hesitant to children's vaccines (85% times more) compared to the never-married people on average (p-value=0.019). People's educational level did not converge significantly on their perceived hesitance toward children's vaccinations (p-value=0.055), neither did their household income correlate significantly with their hesitance toward children's vaccinations (p-value=0.227), but people residing in the Saudi Arabian central region provinces were found to be significantly less predicted (29.4% times less) to be hesitant toward children's vaccinations as compared to people residing in other provinces or regions on average (p-value=0.008). Moreover, the multivariable analysis findings showed that the number of kids in the family had correlated positively with people's hesitance toward children's vaccines, people with a bigger number of children in their family were found to be significantly statistically more predicted to be child-vaccination hesitant in general (OR=1.080, p-value=0.034). People who advised that they had refused the monkeypox vaccine were found to be significantly more hesitant (34% times more) to children's vaccinations compared to people who were hesitant or accepting of monkeypox vaccines (p-value=0.004), also people who refused flu vaccines were found to be significantly more reluctant to children's vaccines (25% times higher) compared to people who are not hesitant to flu vaccines or those who accepted it (p-value=0.050). However, people who refused the meningococcal vaccines were found to be significantly less reluctant to children's vaccines (46.6% times less) as compared to people who were hesitant to the meningococcal vaccines on average (p-value=0.001).

**Table 5:** Multivariable GEE logistic binary regression analysis of people's hesitance toward children's vaccinations GEE: generalized estimating equations

Parameter	Multivariable Adjusted Odds Ratio	95% CI for OR		
		Lower	Upper	p-value
Who answered	1.088	0.889	1.332	0.415
Age of respondent >=45 years	0.765	0.662	0.885	<0.001
Evermarried	1.850	1.107	3.092	0.019
Educational level	0.874	0.761	1.003	0.055
Households income	0.845	0.644	1.110	0.227
Residence province = Central Region	0.706	0.544	0.915	0.008
Number of children in the family	1.080	1.006	1.159	0.034
Refused the monkeypox vaccine	1.340	1.100	1.632	0.004
Refused the flu vaccine	1.280	1.000	1.639	0.050
Refused the meningococcal vaccine	0.534	0.366	0.780	0.001
Worry about vaccine side effects	1.432	1.074	1.910	0.014
Heard or read bad information on social media about vaccines	1.391	1.056	1.831	0.019
Vaccine is not necessary	1.869	1.392	2.509	<0.001
Not safe	1.571	1.006	2.454	0.047
Bad experience from previous vaccine shots	1.721	1.111	2.665	0.015
Did not take COVID vaccines yet	1.622	1.214	2.166	0.001
Constant	0.690			0.503
Dependent variable: children's vaccination hesitance (Low/High)				

## Discussion

Our survey revealed that the prevalence of parents' vaccine hesitancy was 374 (30.9%) while the overall percentage of unvaccinated children among the participating parents was 282 (23.2%). Interestingly, we observed a trend toward a negative association between educational level and hesitancy status, whereas vaccination status was significantly associated with the age of the youngest child under care and the age of the participants. Despite collecting the data during the Mpox WHO alert, the Mpox vaccine had the highest refusal rate at 345 (28.5%) while the COVID-19 vaccine had the highest hesitancy rate at 352 (29.1%). Even though the general KSA public worried more about COVID-19 than Mpox at the time, they still expressed a high degree of hesitancy [14]. Regarding the reasons for hesitancy, we identified that the most impactful factor was exposure to negative media content, affecting 449 (18.98%) of participants, which was closely followed by concerns about vaccine safety, affecting 379 (16.02%) of parents. These findings shed light on the complex factors influencing parental vaccine hesitancy and underscore the importance of addressing misinformation and safety concerns to promote vaccination rates and safer public health, as was concluded from other studies in KSA [15,16].

A study conducted by Alsubaie et al. [5] just before the COVID-19 pandemic in KSA found that the prevalence of vaccine hesitancy among parents was (20%) while in our research, we found that the prevalence was 374 (30.9%). According to these findings, it seems that the pandemic has increased hesitancy by almost one-half in the same population. A 2021 study conducted in Saudi Arabia showed that 24% of parents rejected the vaccine schedule assigned by the Saudi Ministry of Health and 17% expressed reluctance [6]. The Centers for Disease Control and Prevention (CDC) in the U.S. reported that while overall childhood immunization rates remained high between 2012 and 2017, there continued to be an increase in the number of children who had received no vaccines at 24 months [17]. More recently, in March 2020, an assessment of the latest CDC National Immunizations Survey data found that more than one-third of U.S. children between the ages of 19 and 35 months were not following the recommended early childhood immunization schedule [17].

When looking into the reasons for vaccine hesitancy, most parents were hesitant because of exposure to negative and misleading information on social media (18.98%) like WhatsApp or Twitter, which are the most common platforms used in KSA for medical information sharing [18]. Vaccine hesitancy is driven by various factors, including conspiracy theories, general distrust, belief in alternative treatments, and safety concerns [19]. As compared to the pre-COVID-19 era, negative information on social media became a larger factor negatively affecting parental perceptions about vaccination [12]. The second most common concern was fear of the side effects/safety. By examining the two most common concerns, we can see that safety is the main reason for the hesitancy. Other studies have investigated the factors behind vaccine hesitancy, with one study in KSA identifying the potential side effects of the vaccine as the most common concern [16,20]. Another study was conducted in the United Arab Emirates before the COVID-19 era and found that the parent’s greatest concerns were mainly the side effects and safety. Another study suggested that children whose parents are hesitant or have concerns about vaccines can obtain vaccination coverage that is comparable to children whose parents have no vaccine safety concerns if the parent’s decision to get their child immunized is influenced by a trusted healthcare provider, especially their physician [21].

A study done in 2015 showed many factors that contribute to hesitancy toward vaccination, such as knowledge and information sources, experiences with vaccination and vaccine-preventable diseases (VPDs), the role of health professionals and their recommendations, as well as the role of the public health system, trust, and social norms [22]. In our study, we did not find new factors contributing to vaccine hesitancy during the COVID-19 era. Surprisingly, the least anticipated factors were found to be associated with the leading concerns toward vaccine hesitancy. For instance, we discovered that the influence of media content on parents’ decision to withhold vaccines for their children was unexpectedly significant. This result highlights the need for increased awareness and education on social media platforms to counteract the rapid spread of misleading information among parents [23].

In our study, we observed that educational level did not show a statistically significant association with vaccination and hesitancy status. However, in another study by Alsubaie et al. [6], they reported a statistically significant association (p<0.001) between educational level and vaccine hesitancy. Specifically, parents with postgraduate degrees, such as Masters or PhD degrees, were found to be more vaccine-hesitant compared to parents with Bachelor's degrees or school degrees. The differing findings between our study and the Alsubaie et al. study may be attributed to variations in study populations, methodologies, or contextual factors that warrant further investigation.

Indeed, understanding the complexities of science and keeping up with the constantly evolving vaccine guidelines can be challenging for some individuals. Such individuals might be more susceptible to emotional appeals lacking factual basis on social media [24]. Therefore, there is a pressing need for proactive measures, including the creation of reliable and easily accessible social media content tailored to parents' cultural backgrounds, to transparently address their vaccine-safety concerns, emphasizing the importance and efficacy of childhood vaccines [23]. By offering parents scientifically grounded information, we can improve their understanding and confidence in vaccines, which may help reduce vaccine hesitancy [25].

Before the COVID-19 era, parental vaccine hesitancy centered around various vaccinations, such as influenza, measles, and HBV, among others [11,12,26,27]. However, during the COVID-19 pandemic, we observed a shift in hesitancy patterns, with parents being most hesitant toward the COVID-19 vaccine while the Mpox vaccine experienced the highest rejection rates, indicating hesitancy toward newly developed vaccines. A study published in 2018 also highlighted how people’s fears of the potential outcomes and side effects of vaccines can lead to hesitancy or rejection unless they are assured of their safety [28]. In our study, we found that influenza was the fourth most hesitant vaccine for parents, following COVID-19 and the updated COVID-19 omicron BA.4 and BA.5 vaccine. Surprisingly, the Hemophilus influenza b (HIB) vaccine ranked sixth in hesitancy. Another study conducted in KSA showed similar results, indicating that parents were more hesitant toward COVID-19 vaccines compared to other scheduled childhood ones [6,29]. Thus, the COVID-19 pandemic has had a notable influence on parents, making them more hesitant toward vaccines, particularly toward the COVID-19 vaccines compared to other childhood ones. To address parental vaccine hesitancy, we recommend health facilities implement educational programs focused on COVID-19 and other vaccines, which aim to enhance parents’ knowledge regarding the importance of vaccinating their children [30].

Limitations and future research

This study has certain limitations that should be acknowledged. First, the use of snowball sampling may have introduced sampling bias, potentially affecting the generalizability of the study’s findings. Second, as with any survey-based research, the information provided by the parents may be subject to recall bias, and the experiences may not be fully captured. Additionally, relying only on self-reported data without cross-referencing with medical records might lead to inaccuracies in vaccination status. Other limitations include the reliance on self-reported parental responses, which may not accurately reflect actual vaccination practices at later stages. Moreover, as the data were collected in 2022, it captures a specific post-COVID-19 pandemic period.

Future research should consider alternative sampling methods that minimize bias and enhance the representativeness of the study population. Incorporating data from medical records would provide more accurate information regarding vaccination status. Additionally, future studies could focus on implementing educational campaigns aimed at parents. These campaigns could raise awareness about vaccine-preventable diseases, their associated risks, and the benefits of adhering to scheduled childhood vaccinations. By increasing parents' understanding and knowledge, we can strive to reduce vaccine hesitancy and promote vaccination rates, contributing to improved public health outcomes. Future research is warranted to explore the evolution of vaccine hesitancy trends over a longer duration to assess how attitudes and behaviors may change over time and in response to ongoing public health interventions.

## Conclusions

Our study highlights the presence of parental vaccine hesitancy during the COVID-19 era, with significant hesitancy observed toward the COVID-19 vaccine and a high rejection rate for the Mpox vaccine. We found that educational level was not statistically significant in predicting hesitancy status, suggesting that other factors may be more influential in shaping parental attitudes toward vaccination. These findings also highlight the importance of addressing misinformation and concerns surrounding vaccine safety and efficacy. Public health efforts should focus on providing accurate and easily accessible information through educational campaigns on social media and other healthcare platforms such as official Ministry of Health websites. Additionally, incorporating data from medical records in future studies could enhance the reliability of findings and provide a more comprehensive understanding of vaccination status and hesitancy trends.
